# Comparison of the clinical outcomes of percutaneous vertebroplasty vs. kyphoplasty for the treatment of osteoporotic Kümmell’s disease:a prospective cohort study

**DOI:** 10.1186/s12891-020-03271-9

**Published:** 2020-04-13

**Authors:** Jian-Zhong Chang, Ming-Jian Bei, Dong-Ping Shu, Cheng-Jun Sun, Ji-Bin Chen, Ya-Ping Xiao

**Affiliations:** 1 Department of Orthopedic Surgery, CR & WISCO General Hospital, Wuhan University of Science and Technology, No. 209 Yejin Road, Wuhan, 430000 Hubei Province People’s Republic of China; 2 Department of Orthopedic Surgery, Emergency General Hospital, Beijing, 100028 People’s Republic of China; 3 Department of Orthopedics, Wuhan Hanyang Hospital, Wuhan University of Science and Technology, Wuhan, 430050 People’s Republic of China

**Keywords:** Kümmell’s disease, osteoporosis, Vertebral compression fracture, Percutaneous vertebroplasty, Percutaneous kyphoplasty

## Abstract

**Background:**

Percutaneous vertebroplasty (PVP) and percutaneous kyphoplasty (PKP) are widely used in the treatment of Kümmell’s disease. The purpose of this article is to investigate the clinical efficacy of PVP and PKP for Kümmell’s disease.

**Methods:**

The clinical data that 56 cases of Kümmell’s disease treated with either PVP (28 cases) or PKP (28 cases) from December 2015 to December 2017 were prospectively analyzed. Gender, age, course of disease, injury segment, bone mineral density (BMD), visual analogue scale (VAS), Oswestry disability index (ODI), imaging measurement indexes before surgery between the two groups showed no significant difference (all *P* > 0.05). The bone cement leakage rate, bone cement injection amount, operation time, VAS, ODI, the rate of vertebral compression, correction rate of kyphosis and refracture rate of adjacent vertebra in 2 years were compared between the two groups to calculate clinical efficacy.

**Results:**

The two groups were followed up for 24–48 months. There was no significant difference in the follow-up time, amount of bone cement injected, incidence of bone cement leakage and refracture rate of adjacent vertebrae between the two groups (all *P* > 0.05). The operation time, intraoperative blood loss and fluoroscopy times of the PVP group were significantly lower than those of the PKP group (all *P* = 0.000). VAS score and ODI of the two groups were significantly lower at 1 day, 1 year and 2 years after surgery than before surgery (all *P* < 0.05), but there was not statistically significant difference between the two groups at each time point after surgery (all *P* > 0.05). The rate of vertebral compression and kyphosis correction in the two groups were significantly corrected (*P* < 0.05, respectively) and decreased significantly with time (all *P* < 0.05), But there was not significant difference between the two groups at any time point (all *P* > 0.05).

**Conclusion:**

Both PVP and PKP can achieve similar effects in the treatment of Kümmell’s disease. Because the cost, operation time, blood loss, radiation exposure and surgical procedure of PVP are less than those of PKP, PVP has more clinical priority value.

## Background

Kümmell’s disease is a special type of osteoporotic vertebral compression fracture (OVCF), which is relatively rare in clinical practice [1]. Kümmell’s disease is also known as nonunion after OVCF, delayed vertebral osteonecrosis after trauma, and delayed vertebral collapse [2, 3]. Imaging examination can discover the late-onset collapse of vertebrae and characteristic change of intravertebral vacuum cleft (IVC), which is considered one of important inducing factors for injured vertebral progress after the collapse, deformity of kyphosis, intractable back pain and spinal cord damage after OVCF [4].

With the widespread development of percutaneous vertebroplasty (PVP) and percutaneous kyphoplasty (PKP) in the treatment of OVCF, many patients seek PVP or PKP treatment in order to restore the stability of the injured vertebrae and relieve low back pain [5, 6]. Therefore, the literature on the use of PVP or PKP to treat Kümmell’s disease has gradually increased, whose results showed that PKP and PVP can achieve good clinical results in treating this disease [7]. However, the selection criteria for PVP and PKP treatment are inconclusive at present.

Therefore, we conducted a comparative study on Kümmell’s patients who received either PVP or PKP treatment in our hospitals from December 2015 to December 2017 to investigate the clinical efficacy, advantages and disadvantages of the two surgical methods, and to provide a reference for clinical selection of treatment methods.

## Methods

### Selection criteria

This study is a prospective, randomized, controlled study. The patients were blinded, but the researchers were not fully blinded. The inclusion criteria are as follows: ① Patients suffered single-segment Kümmell’s disease without new OVCF in the adjacent vertebrae; ② Computed tomography (CT) or magnetic resonance imaging (MRI) examination confirmed the existence of IVC. the IVC refers to a significant radiolucency (gas containing), which is located centrally or adjacent to the vertebral body endplates as seen on CT or plain radiographs. On MRI, it is usually shown as a low signal intensity on a T1-weighted image and as a high signal or a low signal on a T2-weighted image, depending on whether the fluid or gas fills the cleft [4]; ④ Dual energy X-ray measurement of bone mineral density (BMD) T value was less than − 2.5; ⑤ PVP or PKP was conducted with bilateral pedicle; ⑥ Follow-up time was more than 2 years; ⑦ Postoperative calcium supplementation and anti-osteoporotic drugs were applied; ⑧ No fall or other trauma occurred. Exclusion criteria are as follows: ① Patients with severe cardiopulmonary dysfunction; ② Patients with coagulopathy; ③ Patients with local or systemic infection; ④ Patients with nerve root or spinal cord compression symptoms; ⑤ Patients with pathological fractures other than osteoporosis.

### Baseline data

**Fig. 1:**
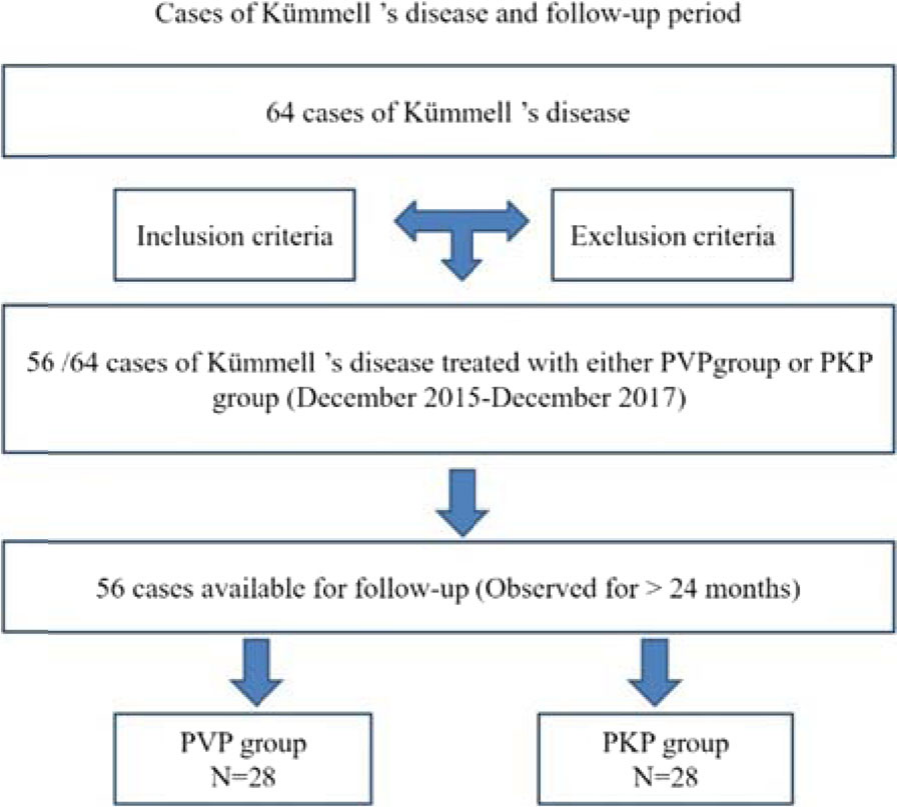
Cases of Kümmell ‘s disease and follow-up period

From December 2015 to December 2017, 56 of the 64 patients with Kümmell’s disease met the selection criteria for inclusion in the study, of which 28 cases received PVP treatment (PVP group) and 28 cases received PKP treatment (PKP group) (Fig. 1). This study was approved by the ethics committees of authors’ hospitals. All patients with clinical data and pictures gave written informed consent.

The PVP group included 6 males and 22 females, aged 63–85 years, with an average of 75.0 ± 5.8 years. The course of disease was 2–14 months, with an average of 8.7 ± 3.0 months. Twenty cases had no obvious history of trauma, and 8 cases had a history of falls. Injured vertebra segments included 1 case in T9, 1 case in T10, 5 cases in T11, 8 cases in T12, 8 cases in L1, 2 cases in L2, 2 cases in L3, and 1 case in L4. The dual energy X-ray BMD T value was − 2.83 - -6.13, and the average value was − 4.35 ± 0.91.(Table 1).

**Table 1:** Baseline data of the two groups

Parameter	PVP group	PKP group	t/χ^2^	*P*
Cases	28	28		
Gender				
Male (cases)	6	8	0.381	0.537
Female (cases)	22	20		
Age (years)	75.0 ± 5.8	75.1 ± 5.7	−0.632	0.533
Course of disease (months)	8.7 ± 3.0	8.4 ± 2.9	0.960	0.338
Fall history				
Yes (cases)	8	10	0.327	0.567
No (cases)	20	18		
BMD (T value)	- 4.35 ± 0.91	−4.47 ± 0.89	−1.024	0.307

The PKP group included 8 males and 20 females, aged 62–86 years, with an average of 75.1 ± 5.7 years. The course of disease was 2–13 months, with an average of 8.4 ± 2.9 months. Eighteen cases had no obvious history of trauma, and 10 cases had history of falls. Injured vertebral segment included 2 cases of T9, 3 cases of T11, 7 cases of T12, 9 cases of L1, 4 cases of L2, and 1 case of L3 and 2 cases of L4. The dual energy X-ray BMD T value was − 2.54 - -5.91, and the average value was − 4.47 ± 0.89.(Table 1).

Gender, age, course of disease, injury segment, BMD, VAS, the Oswestry disability index (ODI) and imaging indexes of the two groups were comparable (all *P* > 0.05, Tables 1, 2 and 3).

**Table 2:** Comparison of clinical efficacy between the two groups

Parameter	PVP group	PKP group	t/χ^2^	*P*
Follow-up time (months)	35.3 ± 6.99	35.2 ± 7.63	0.115	0.909
Amount of bone cement injected (ml)	4.2 ± 1.15	4.6 ± 1.55	−1.875	0.065
Operation time (min)	34.8 ± 3.47	45.1 ± 5.15	−56.032	0.000
Intraoperative blood loss (ml)	16.9 ± 3.49	21.2 ± 5.29	−15.479	0.000
Fluoroscopy times	15.8 ± 3.50	20.5 ± 4.16	−19.060	0.000
Bone cement leakage				
Yes (cases)	5	3	0.163	0.686
No (cases)	24	25		
VAS scores				
Before surgery	8.0 ± 0.77	8.0 ± 0.75	0.698	0.486
At 1 day after surgery	2.8 ± 0.75*	2.7 ± 0.81*	0.301	0.764
At 1 year after surgery	2.4 ± 0.70*	2.6 ± 0.84*	−1.398	0.165
At 2 years after surgery	2.5 ± 0.70*	2.5 ± 0.84*	−0.356	0.722
ODI				
Before surgery	84.5 ± 5.94	84.9 ± 8.23	−2.293	0.062
At 1 day after surgery	29.0 ± 7.62*	30.4 ± 7.73*	−3.533	0.056
At 1 year after surgery	29.8 ± 7.02*	29.9 ± 7.01*	−0.407	0.684
At 2 years after surgery	29.9 ± 7.11*	31.0 ± 7.56*	−2.872	0.054
Adjacent vertebral fractures				
Yes (cases)	2	3	0.220	0.639
No (cases)	26	25		

*Compared to before surgery, *P* = 0.000

**Table 3:** Comparison of two groups of imaging data

Parameter	PVP group	PKP group	t/χ^2^	*P*
Rate of vertebral compression				
Pre-operation	0.74 ± 0.11	0.75 ± 0.12	−0.088	0.930
At 1 day after operation	0.84 ± 0.11*	0.86 ± 0.09*	−0.515	0.609
At 1 year after operation	0.82 ± 0.13	0.82 ± 0.09	0.248	0.805
At 2 years after operation	0.79 ± 0.15	0.80 ± 0.11	−0.290	0.773
Correction rate of kyphosis				
At 1 day after operation	31.0 ± 6.34	32.6 ± 6.19	−2.467	0.053
At 1 year after operation	30.0 ± 6.24#	29.9 ± 5.96#	0.093	0.926
At 2 years after operation	28.8 ± 6.37#&	28.7 ± 6.59#&	0.509	0.611

* Compared to pre-operation, *P* < 0.05

# Compared with 1 day after operation, *P* < 0.05

& Compared with 1 year after operation, *P* < 0.05

### Surgical procedures

**Fig. 2:**
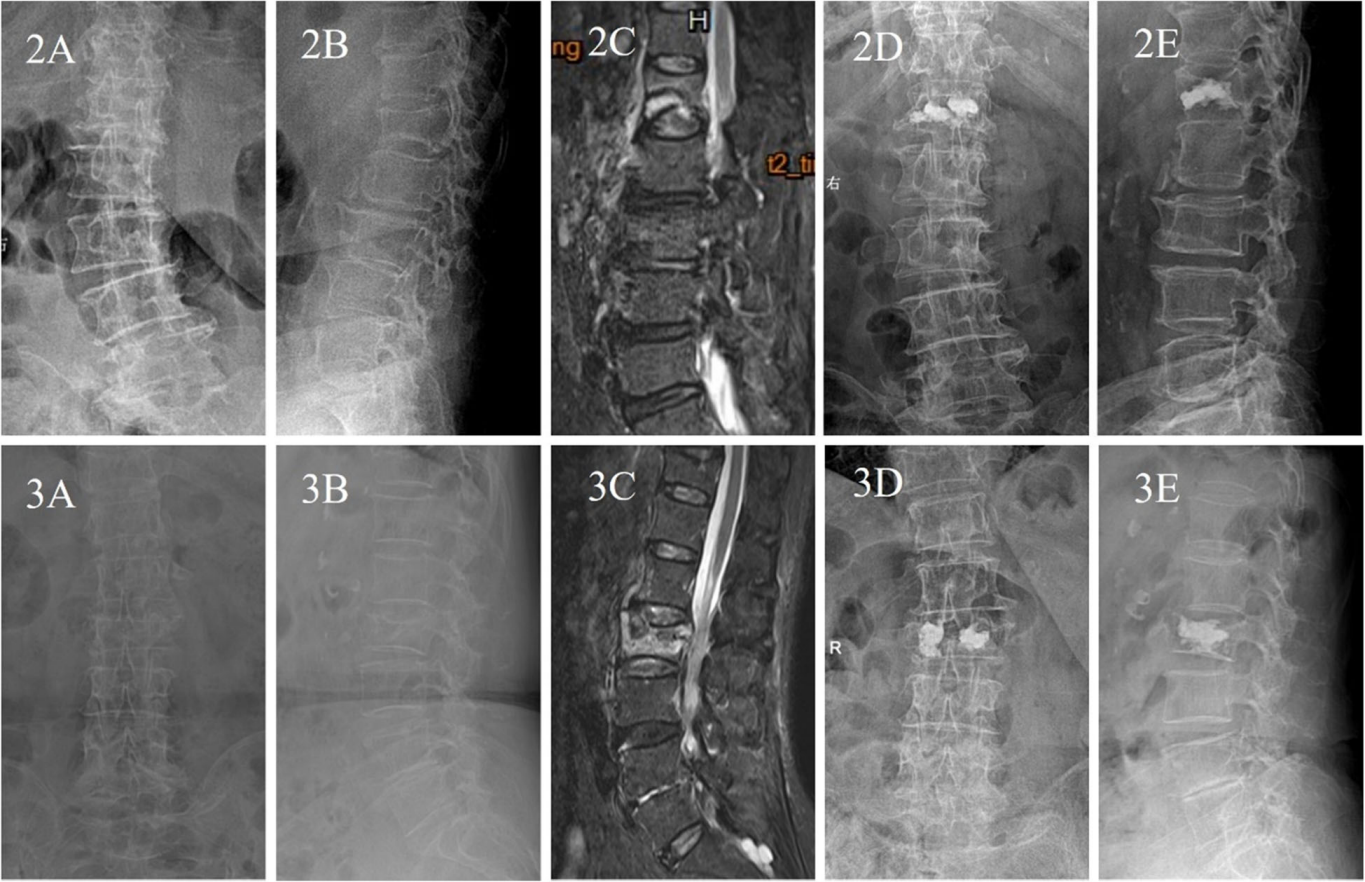
A 75-year-old female patient with Kümmell’s disease at L1 in PVP group:2A Anteroposterior X-ray film before operation; 2B Lateral X-ray film before operation; 2C MRI image before operation; 2D Anteroposterior X-ray film after operation; 2E Lateral X-ray film after operation. 3. A 83-year-old female patient with Kümmell’s disease at L2 in PKP group: 3A Anteroposterior X-ray film before operation; 3B Lateral X-ray film before operation; 3C MRI image before operation; 3D Anteroposterior X-ray film after operation; 3E Lateral X-ray film after operation

Both groups of surgeries were performed by the same group of physicians, and the PVP and PKP procedures followed the same principle (Fig. 2). The patients were placed in the prone position to maintain the spine’s posterior extension for position reduction. Bilateral pedicle puncture with local anesthesia guided by C-arm X-ray machine. PVP procedure: the working sleeve reached the IVC area in the anterior 1/3 of the vertebra or close to the IVC area. Polymethylmethacrylate (Tecres SPA, Verona, Italy) was injected into the IVC region using a 3.5 mm side opening bone cement injector (Shanghai Kinetic Medical Co., Ltd., Shanghai, China) until the entire crack was fully filled and the cement was well dispersed. PKP procedure: after the working sleeve reached the IVC area, the extended balloon catheter (Shanghai Kinetic Medical Co., Ltd., Shanghai, China) was first delivered to the IVC area through the work channel. The balloon was expanded, and the pressure of the balloon system was controlled within 15 atm to make the satisfactory reduction of the fractures around the IVC area and the corresponding endplate. Finally, the bone cement was injected into the IVC region through the side opening injector until the entire fracture was filled.

### Postoperative management

The patient remained in a supine position for 8–12 h after the operation. Patients were still confined to bed rest. Lumbar and back braces were used to protect the patient from bed during 1–2 months after the operation. Oral bisphosphonates, vitamin D and calcium tablets and other drugs were used for anti-osteoporosis treatment.

### Outcome measurement

Clinical efficacy: the leakage rate of intraoperative bone cement, injection amount of bone cement, and operation time of the two groups were recorded. VAS score was used to evaluate the pain degree of low back [8]. The ODI was used to evaluate the severity of the dysfunction [9]. The incidence of new adjacent vertebral fracture was recorded in 2 years.

Imaging measurement indexes: the height of the injured vertebral body and the kyphosis angle were measured before and after the operation by the anteroposterior and lateral X-ray images [10]. And the following indexes were calculated. 1) Rate of vertebral compression = the height of the injured vertebral body / the average height of adjacent upper and lower vertebral body heights [10]; 2) Correction degree of kyphosis = (preoperative kyphosis angle - postoperative kyphosis angle) / preoperative kyphosis angle × % [11].

### Statistical analysis

SPSS19.0 statistical software (IBM Corp., Armonk, NY, USA) was used for statistical analysis. Measurement data were expressed by mean ± standard deviation. The Levene test was used to test the homogeneity of variance. The independent sample t test and the One-Way ANOVA (Bonferroni or Dunnett T3) were used for inter-group comparisons at different time points. The repeated measures ANOVA was used for intra-group comparisons at different time points. The counting data were tested with χ^2^ test. Test level α was 0.05.

## Results

### Clinical outcomes

The two groups were followed up for 24–48 months. The follow-up time and intraoperative amount of bone cement injection between the two groups was not significant difference (*P* > 0.05, respectively). The operation time, intraoperative blood loss and fluoroscopy times of the PVP group were significantly lower than those of the PKP group (all *P* = 0.000). Bone cement leakage occurred in 5 cases (17.9%) of the PVP group, including 2 cases of anterior wall, 1 case of upper intervertebral space, 1 case of lower intervertebral space and 1 case of lateral wall. Bone cement leakage occurred in 3 cases (10.7%) of the PKP group, including 1 case of anterior wall, 1 case of upper intervertebral space and 1 case of lateral wall. But no related clinical symptoms occurred in both groups. There was not significant difference in the number of incidence of bone cement leakage between the two groups (*P* > 0.05). (Table 2).

VAS score and ODI of the two groups were significantly lower at 1 days, 1 year and 2 years after surgery than before surgery (all *P* < 0.05), and there was not statistical difference between the two groups at each time point after surgery (all *P* > 0.05). During the 2 years of follow-up after the operation, 2 cases (7.1%) of the PVP group and 3 cases (10.7%) of the PKP group had adjacent vertebral fractures, and the difference between the two groups was not statistical difference (*P* > 0.05). (Table 2).

### Imaging evaluation outcomes

The correction rate of kyphosis and rate of vertebral compression in the two groups were significantly corrected (*P* < 0.05, respectively) and gradually decreased significantly with time (all *P* < 0.05) (Tables 2 and 3), which indicates that the height of vertebral body and kyphosis angle were significantly recovered and gradually lost after surgery. These indexes between the two groups at any time point was not significant difference (all *P* > 0.05, Table 3).

## Discussion

We found that follow-up time, incidence of bone cement leakage, refracture rate of adjacent vertebra and intraoperative amount of bone cement injection between the two groups was not statistical difference. Both groups significantly relieved patients’ pain of low back, recovered the height of vertebral body and kyphosis angle and improved their quality of life, but PVP was associated with less surgical time, blood loss, and radiation exposure than those of PKP. The rate of vertebral compression and kyphosis correction between the two groups was not found significantly difference, but decreased significantly with time, which suggests that the correction was gradually lost.

Since 1984, Galiber et al. [12] applied PVP in the treatment of 1 case of C 2 vertebra invasive hemangioma to find its advantages of simple operation and definite effect. PVP and PKP have gradually become one of the effective methods to treat vertebral tumor and OVCF [8, 13, 14]. Kümmell’s disease is a rare type in OVCF after minor trauma, which is progressive occurrence of vertebral collapse and kyphosis due to vertebral ischemia and necrosis. Kümmell’s disease is often manifested as intractable pain of low back, pseudojoint formation, and may be accompanied by nerve injury in severe cases [15, 16]. In plain radiographs and CT imaging of vertebral body pseudoarthrosis, the fracture sites often present gas accumulation, which is termed the vacuum phenomenon or IVC sign. Vacuum phenomena often appear as intravertebral radiolucent shadows that are typically band like or linear in shape and are often accompanied by peripheral sclerosis [17]. MRI imaging can find limited fluid filling in the vertebral body. So Kümmell’s disease is also referred to as delayed post-traumatic vertebral collapse disease, nonunion of vertebral fractures, and vertebral ischemic necrosis [1, 2, 18]. Previous studies have suggested that IVC is mainly located in the thoracolumbar region [4, 19], which is similar to the segmental distribution of the two groups of patients in this study. 87.5% of the fractured vertebral bodies were located in the thoracolumbar segment, most of which were wedge fractures. And the fissures mostly occurred near the upper or lower endplate of the vertebral body. These suggests that the occurrence of the disease may be related to the repeated stress activity and high mobility in the thoracolumbar segment, leading to vertebral nonunion and ischemic necrosis.

Previous studies have found that a small dose of bone cement can restore the mechanical properties of the fractured vertebral body, and the injection amount of bone cement has no significant correlation with the analgesic effect, even believed that 1.5 ml of bone cement is injected into each vertebral body to obtain satisfactory analgesic effect [20, 21]. Biomechanics studies in vitro have confirmed that the strength of the vertebral body can be restored by injecting about 2 ml bone cement or 16% of the vertebral volume with bone cement, and the stiffness of the vertebral body can be restored by injecting about 4 ml bone cement or 24% of the vertebral volume with bone cement [21]. In this study, the average injection amount of bone cement was 4.2 ± 1.15 ml in the PVP group and 4.6 ± 1.55 ml in the PKP group, both of which met the requirements of restoring the strength and stiffness of the vertebral body without significant difference between the two groups.

Significant pain relief was observed after surgery in both groups, and maintained until the last follow-up, However, no significant difference was seen between the groups. After filling the fissures in the vertebral body with bone cement, the height and kyphosis deformity of the vertebral body were partially restored and corrected, and the abnormal activity of the injuried vertebral body was eliminated, which was an important reason for pain relief [11]. Previous studies have found that both PVP and PKP can effectively relieve the lower back pain of Kümmell’s disease, achieve satisfactory clinical effect, and partially restore the height of the vertebra and correct the kyphosis [11, 22–29]. During spinal flexion and extension, due to the presence of IVC and the formation of pseudojoint, the injured vertebra of Kümmell’s disease can stretch and expand, which can widen the fractured vertebral body. Therefore, the height of the collapsed vertebral body can be retracted and kyphosis has been partially corrected during spinal hyperextension. In PVP treatment, bone cement is usually limited to diffusion in the fissure, which can maintain the effect of hyperextension kyphotic correction without the help of balloon dilation for reduction in PKP treatment. Previous studies have also found that patients with Kümmell’s disease can achieve spontaneous reduction in the postextensor position without further balloon expansion reduction [30, 31]. Heo et al. [32] reported that excessive reduction tends to accelerate the process of vertebral ischemia and necrosis, leading to severe re-collapse. Therefore, excessive reduction of intraoperative injured vertebra should be avoided. We found that the rates of vertebral compression and kyphosis correction both in PVP and PKP groups obtained obvious correction, but no statistical difference of the rates between the two groups was observed, which further confirmed the point of view “ patients of Kümmell ‘s disease can have spontaneous reduction in the posterior extension without further balloon expansion “.

We found that the rates of vertebral compression and kyphosis correction in the two groups gradually decreased significantly with time, suggesting that vertebral height and kyphosis angle gradually lost after surgery, which was consistent with previous findings [7, 14]. In the treatment of OVCF without IVC by PVP and PKP, the injected bone cement of the former is mainly embedded in cancellous bone, while the bone cement of the latter is mainly filled with clumps, so stress occlusion is more likely to occur after PKP and lead to recollapse [33]. However, in this study, there was no significant difference of the vertebral recollapse between the two groups after 2 years of post-operation. The reason was that the distribution of bone cement in the IVC region was inconsistent with that in the non-IVC region. Due to the low pressure in the IVC region and the obstruction of the surrounding fibrous membrane, IVC was used as a “reservoir” during intraoperative injection of bone cement in both groups, and bone cement was filled in the IVC region in the form of solid masses. The limited bone cement mass cannot connect with the adjacent endplates of the upper and lower levels and strengthen cancellous bone of the vertebrae, thus failing to support the normal physiological stress from the body and causing the collapse again [34].

The most common complications of PVP and PKP are bone cement leakage and adjacent vertebral fractures [35, 36]. The incidences of cement leakage of PVP and PKP for OVCF were 54.7 and 18.4%, respectively [36]. Krauss et al. [37] reported that in the treatment of OVCF with IVC by PVP, the bone cement leakage rate was 18.2%. Wang et al. [38] reported that the bone cement leakage rate of OVCF with IVC was 7.4% in PKP treatment. This study found that the leakage rates of bone cement were 17.9 and 10.7% in PVP and PKP group respectively, and both are similar to previous studies, which may be related to accurate preoperatively measurement of surgical approach, careful operation by the operator and not pursuing the maximum amount of bone cement. Besides, kümmell’s disease exists obvious hardened zone and “crack” of vertebral body. A closed space is formed by the fissure, the surfaces of hardened zone, and the anterior longitudinal ligament. The injection of bone cement is confined in the closed space, and less prone to spread to other parts of the vertebral body. Therefore, the abnormal leakage of bone cement both in PKP group and PVP group was lower than that in the treatment of OVCF. In this study, although there was no significant difference of the bone cement leakage rate between the PVP group and the PKP group, the bone cement leakage rate of the PKP group was lower than that of the PVP group. The reason was mainly related to the fact that the PKP group could squeeze the surrounding cancellous bone during balloon expansion and reduce the bone cement leakage. In the other study, among the 219 patients of OVCF with single thoracolumbar fractures, 29 cases (13.2%) occurred non-surgical vertebral fractures in the PKP treatment [39]. Eleven patients (14.1%) in the early PVP group (*n* = 78) and 18 patients (39.1%) in the late PVP group (*n* = 46) experienced an adjacent vertebral fractures during the first year following PVP [40]. In this study, the secondary fracture rates of PVP and PKP were 7.1 and 10.7%, respectively, which were both lower than previous studies, and may be related to postoperative restriction of premature activity, thoracolumbar protection and continuous anti-osteoporosis treatment.

However, this study also had the following limitations. Despite the use of the blinded and random fashion, researchers failed to fully implement the principle of blindness. This was a prospective study with a small number of patients in each group. Furthermore, there is a lack of time biomechanical study on cement distribution in vertebrae to support the results. The current findings require further validation in multicenter, randomized, double-blind clinical trials.

## Conclusion

Both PVP and PKP can achieve similar effects in the treatment of Kümmell’s disease with the advantages of simple operation, short operation time, small trauma, and quick recovery, which can restore the partial height and kyphosis of the fractured vertebral body, quickly alleviate pain and improve the living quality of patients. Because the cost, operation time, blood loss, radiation exposure and surgical procedure of PVP are less than those of PKP, PVP has more clinical priority value.

## Data Availability

The datasets used and/or analysed during the current study are available from the corresponding author on reasonable request.

## Abbreviations

PVP: Percutaneous vertebroplasty
PKP: Percutaneous kyphoplasty
OVCF: Osteoporotic vertebral compression fracture
IVC: Intravertebral vacuum cleft
ODI: Oswestry disability index
VAS: Visual analogue scale
